# Metatranscriptomics Reveals the Diversity of Genes Expressed by Eukaryotes in Forest Soils

**DOI:** 10.1371/journal.pone.0028967

**Published:** 2012-01-06

**Authors:** Coralie Damon, Frédéric Lehembre, Christine Oger-Desfeux, Patricia Luis, Jacques Ranger, Laurence Fraissinet-Tachet, Roland Marmeisse

**Affiliations:** 1 Ecologie Microbienne, UMR CNRS 5557, USC INRA 1193, Université de Lyon, Université Lyon 1, Villeurbanne, France; 2 Pôle Rhône-Alpes de Bioinformatique, Université de Lyon, Université Lyon 1, Villeurbanne, France; 3 Biogéochimie des Ecosystèmes Forestiers, INRA centre de Nancy, Champenoux, France; Universidad Miguel Hernandez, Spain

## Abstract

Eukaryotic organisms play essential roles in the biology and fertility of soils. For example the micro and mesofauna contribute to the fragmentation and homogenization of plant organic matter, while its hydrolysis is primarily performed by the fungi. To get a global picture of the activities carried out by soil eukaryotes we sequenced 2×10,000 cDNAs synthesized from polyadenylated mRNA directly extracted from soils sampled in beech (*Fagus sylvatica*) and spruce (*Picea abies*) forests. Taxonomic affiliation of both cDNAs and 18S rRNA sequences showed a dominance of sequences from fungi (up to 60%) and metazoans while protists represented less than 12% of the 18S rRNA sequences. Sixty percent of cDNA sequences from beech forest soil and 52% from spruce forest soil had no homologs in the GenBank/EMBL/DDJB protein database. A Gene Ontology term was attributed to 39% and 31.5% of the spruce and beech soil sequences respectively. Altogether 2076 sequences were putative homologs to different enzyme classes participating to 129 KEGG pathways among which several were implicated in the utilisation of soil nutrients such as nitrogen (ammonium, amino acids, oligopeptides), sugars, phosphates and sulfate. Specific annotation of plant cell wall degrading enzymes identified enzymes active on major polymers (cellulose, hemicelluloses, pectin, lignin) and glycoside hydrolases represented 0.5% (beech soil)–0.8% (spruce soil) of the cDNAs. Other sequences coding enzymes active on organic matter (extracellular proteases, lipases, a phytase, P450 monooxygenases) were identified, thus underlining the biotechnological potential of eukaryotic metatranscriptomes. The phylogenetic affiliation of 12 full-length carbohydrate active enzymes showed that most of them were distantly related to sequences from known fungi. For example, a putative GH45 endocellulase was closely associated to molluscan sequences, while a GH7 cellobiohydrolase was closest to crustacean sequences, thus suggesting a potentially significant contribution of non-fungal eukaryotes in the actual hydrolysis of soil organic matter.

## Introduction

In terrestrial ecosystems, plant litter degradation is a key ecological feature which controls not only the equilibrium between soil carbon storage and CO_2_ release in the atmosphere, but also the release of essential soil nutrients trapped in dead plant biomass such as organic forms of phosphorus and nitrogen.

In ecological studies, litter degradation is often estimated by measuring parameters such as soil respiration [1], litter mass loss [2] or the activities of specific microbial enzymes in soil extracts [3]. In microbiology, the degradation of plant-derived compounds such as lignocellulose has been studied using a few microbial model species and has recently led to the sequencing of the genomes of different saprotrophic fungal species which use different strategies to degrade plant material [4]–[7], thus revealing the full enzymatic machinery implicated in this process.

Under natural conditions, litter degradation is generally carried out by consortia of species that either act simultaneously or replace one another on a common piece of plant debris in a sometimes predictable manner and not by a single microbial species [8]. It can therefore be anticipated that the molecular machinery deployed to completely mineralize litter in the field is far more complex and diverse than the machinery observed in a single microbial genome. In addition, it is likely that the diversity of this machinery is partly controlled by litter chemistry and complexity and therefore by plant community composition. Indeed, by affecting litter quality and soil physicochemical properties, plant cover could influence microbial community composition and/or diversity [9], [10], and select different microbial taxa that may possess different degradation machineries.

By allowing access to the genome contents of the different microorganisms present in a common environment (metagenomics) or to the set of genes they express (metatranscriptomics), environmental genomics offers a novel opportunity to decipher at the molecular level, complex ecological processes such as plant organic matter degradation, thus bridging the gap between global field measurements and targeted genomic approaches. These approaches have been carried out on a variety of animal digestive systems [11]–[17] or model composts [18] and DNA and/or RNA sequencing of their associated microbial communities has lead to the characterization of numerous members of carbohydrate-active enzyme families whose frequencies differ depending on the origin of the sequenced metagenomic DNA/RNA [11], [15].

Since the primary degradation of lignocellulosic materials in soil is believed to be carried out essentially by fungi, we present a first metatranscriptomic analysis of forest soils focussing specifically on eukaryotic organisms whose polyadenylated mRNA can be separated from prokaryotic and non coding ones, thanks to their poly-A tails [13], [19], [20]. Poly-A mRNA were directly extracted from beech (*Fagus sylvatica*, a broadleaf deciduous angiosperm species) and spruce (*Picea abies*, an evergreen gymnosperm species) forest soils, converted into cDNA then cloned and 10,000 of them per library sequenced using the Sanger technology. The sequence datasets were specifically analysed with respect to the diversity of enzymes implicated in the breakdown of lignocellulosic material and downstream monosaccharide membrane transporters, as well as enzymes and transporters involved in the mobilization of the organic forms of other elements (phosphorus, nitrogen). In addition, since many microbial enzymes produced for soil nutrient acquisition, and/or toxic soil compounds inactivation (e.g. P450 monooxygenases) are also of biotechnological interest, we performed a global evaluation of the sequence dataset with respect to its biotechnological potential.

## Results

### Sequence datasets (Table 1)

**Table 1:** Characteristics of the sequence datasets.

	Spruce soil	Beech soil
No. of sequenced clones	10,000	10,000
No. of contaminating rRNA squences	684	765
No. of “good quality cDNAs”	8606	7905
Average length of cDNA sequences (nucleotides)	482	430
cDNA GenBank/EMBL/DDJB Accession Nos.	FR697056–706058	FR706059–714330
% cDNA in clusters (No. of clusters)	9% (248)	9% (260)
No. of singletons	7851	7222
% cDNA with BLASTX hits (GenBank nr)	48%	39.5%
% cDNA with GO terms	39%	31.5%
% cDNA with GO “Biological process” terms	25.5%	23.5%
% cDNA with E.C. No. (No. of E.C. Nos.)	13% (321)	12% (313)
% cDNA with taxonomic annotation (MEGAN)	46%^d^/50%^s^	37%^d^/42%^s^

d values obtained using the default settings of the MEGAN software (“Min.support” parameter set to five);

s values obtained using the MEGAN software with “Min.support” parameter set to one.

cDNA libraries of ∼9 10^5^ and 5 10^5^ plasmid clones were obtained using poly-A mRNA extracted from beech and spruce soils, respectively. A significant proportion of cDNA inserts were between 400 and 700 bp-long, as estimated by agarose gel electrophoresis (64% and 48% of cDNA for beech and spruce respectively). Following sequencing and cleaning of the sequences (removing bad quality sequences, poly-A tails, vector and adaptor sequences as well as contaminating rRNA sequences, and discarding sequences shorter than 100 nt), 7905 (beech) and 8606 (spruce) cDNAs were retained for further analyses. Contamination levels by eukaryotic and bacterial rRNA sequences represented at most 8.8% of the sequences (beech). These rRNA sequences could have been cloned because they processed A-rich sequence stretches, and therefore may not reflect the taxonomic diversity of the soil microbial communities. Finally the average read length was 430 bp for beech and 482 bp for spruce cDNAs.

For spruce and beech cDNA sequence datasets, 9% of the sequences grouped into clusters which, for more than 70% of them, contained only two sequences (Figure S1 B–C). As a result, the rarefaction curves plotting the numbers of clusters *versus* the numbers of sequences had the shapes of straight lines (Figure S1 A) suggesting that the datasets represented a small proportion of the total metatranscriptome sequence diversities. Only two of the 12 clusters grouping at least 8 sequences corresponded to known genes. One, from the beech library, encoded a fungal hydrophobin, a class of proteins which coat the external surface of hyphae and spores, and the other, from the spruce library, a putative chitinase.

### Taxonomic diversity of the soil eukaryotic communities

For each forest soil, the taxonomic composition of the eukaryotic community contributing to the soil RNA pool was independently estimated using two sequence datasets: (i) 18S rDNA fragments PCR amplified from reverse-transcribed soil RNA (Table S1), and (ii) the sequenced cDNAs. Sequences were assigned to one of eight major eukaryotic phyla, i.e. the Fungi, Metazoas, Plantae, Amoebozoas, Alveolata, Heterokonta, Rhizarias and Excavata (as defined in [21]). The last 5 phyla are collectively referred as the “protists” in the manuscript.

**Figure 1:**
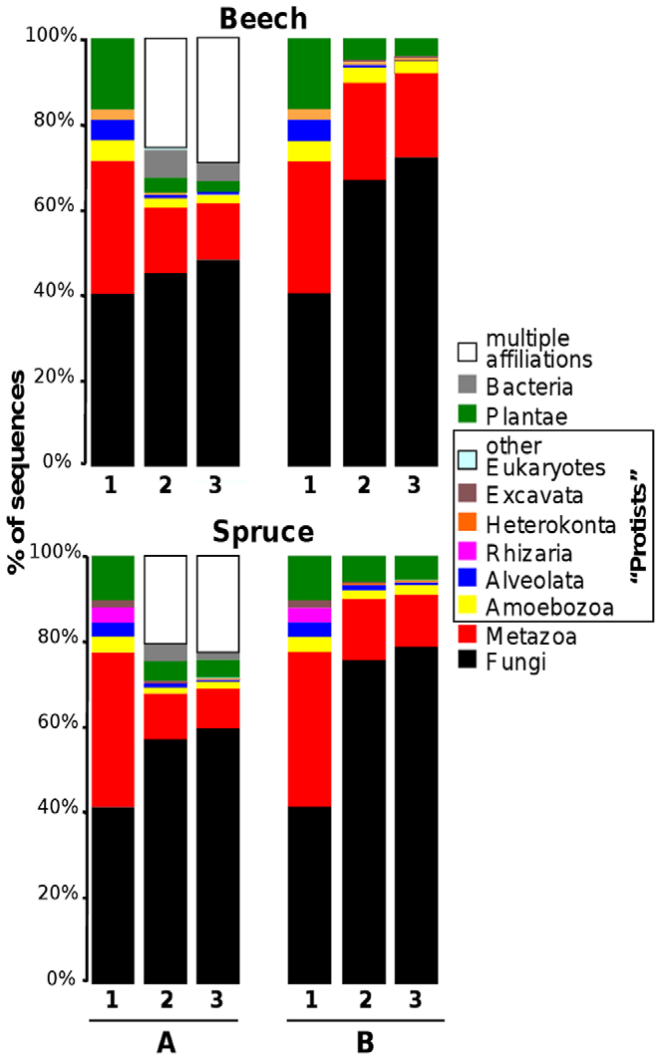
Taxonomic affiliation of the different environmental sequences. PCR amplified 18S rRNA (column 1) and cDNAs (columns 2 and 3) from spruce and beech soils were attributed to one of eight eukaryotic phyla or to the Bacteria. The “multiple affiliation” category contains sequences which could not be unambiguously placed in one of the other categories. (A), Results for all sequences which had a homolog in the GenBank/EMBL/DDJB database; (B), same analysis after removing cDNA sequences from bacteria and cDNA with uncertain “multiple affiliation” for a direct comparison between 18S rRNA and cDNA datasets. For the annotation of cDNA sequences using MEGAN, two different parameters were used; the “stringent analysis” (column 2) corresponded to the “Min Support” parameter set to default value of 5 which was set to 1 in the “less stringent” analysis (column 3).

Whatever the sequence dataset and the studied soil, the Opisthokont group (i.e. essentially Fungi and Metazoas) dominated, representing 71% (beech) and 77% (spruce) of the unambiguously annotated PCR amplified 18S rDNA and 60–61% of the annotated cDNAs (Figure 1 and Table S2). Furthermore, among the Opisthokonts, fungal sequences were the most abundant, representing between 75 and 87% of the cDNAs attributed to this group.

Sequences from each “protist” group were identified at least once in each of the different beech or spruce sequence datasets and protist sequences contributed to the different datasets between 3% (cDNAs) and 12% (amplified 18S rDNA) of the unambiguously annotated sequences. It should however be stressed that the use of a single primer pair to amplify eukaryotic rDNA genes is known to capture only a fraction of the true diversity [22] and that amplification of 18S sequences from several groups of unicellular eukaryotes requires group-specific primers [23].

Using the default parameters and the BLASTX results against the NCBI-nr protein database as input file, the MEGAN software attributed a taxonomic annotation to 46% (spruce) and 37% (beech) of the cDNAs. Among these annotated cDNAs, only 2.5% were attributed to “protists” and 22–29% could not be unambiguously attributed to a single taxonomic group (multiple affiliation category, Figure 1). We asked if under-representation of specific taxonomic groups, such as the protists, and a high prevalence of sequences with multiple potential affiliations may have resulted from the settings of the MEGAN software. Indeed, using default parameters, MEGAN could take into consideration a too high number of BLAST hits (a maximum of five) to infer a taxonomic affiliation thus underestimating the abundance of taxonomic groups poorly represented in sequence databases. This hypothesis was tested by lowering the “Min Support” parameter from 5 to 1 (relaxed setting). Although this modification increased the number of sequences attributed to protists by 50% (which still represented at most 3.5% of the cDNAs); this did not change the overall taxonomic distribution of the cDNAs (Figure 1).

### Global functional annotation of the cDNAs

Using an e-value threshold of 10^−6^, 48% (spruce) and 39.5% (beech) cDNA sequences returned a positive hit in BLASTX searches against the GenBank nr protein database. Among the sequences with no homolog in this preliminary analysis, an additional 227 (spruce) and 238 (beech) contained a registered protein motif identified using InterProScan.

Using Blast2GO, a Gene Ontology (GO) term (associated to either a biological process, a molecular function or a cellular component GO category) could be assigned to 31.5% (beech) and 39% (spruce) of the cDNAs. The five most abundant “biological process” and “molecular function” GO categories were the same for the beech and spruce datasets and altogether accounted for respectively 44% and 43% of the sequences annotated with either a “biological process” and “molecular function” GO term (Figure S2). The two most abundant categories were related to protein synthesis (ribosome biogenesis and translation) and contained, for example, ribosomal proteins encoding sequences.

Finally, 12–13% of the sequences in each datasets encoded proteins with enzymatic activities to which an Enzyme Commission (E.C.) number could be associated (Table 1). Two hundred different E.C. numbers were placed into 117 (spruce) and 112 (beech) different KEGG pathways. Several of these pathways such as glycolysis, tricarboxylic acids (TCA) cycle, nitrogen and sulfur metabolisms, amino acid biosynthesis or degradation (Table 2) are essential to appreciate basic soil processes such as global microbial activities and soil nutrient assimilation. For several of these pathways we identified key enzymes that represent the obligate entry points of major soil nutrients in fungal metabolism (Table S3). This is for example the case of the glutamine synthase (E.C. 6.3.1.2)/NADPH-glutamate dehydrogenase (E.C. 1.4.1.4)/glutamate synthase (E.C. 1.4.1.13) for ammonium; the adenylyl-sulfate kinase (E.C. 2.7.1.25) and the sulfate adenylyltransferase (E.C. 2.7.7.4) for sulfate and aldehyde reductase (E.C. 1.1.1.21) for pentoses (xylose, arabinose) which are end products of hemicelluloses hydrolysis (Table S3).

**Table 2:** A list of KEGG pathways relevant to soil nutrient (C, N, S) utilisation and microbial metabolism for which different cDNA sequences from the spruce and beech soil could be affiliated.

	No. of cDNAs	
KEGG pathway	Beech	Spruce
Nitrogen metabolism	13 (16.4)	17 (19.7)
Alanine, aspartate and glutamate metabolism	19 (24.0)	26 (30.2)
Arginine and proline metabolism	20 (25.3)	22 (25.6)
beta-Alanine metabolism	6 (7.6)	13 (15.1)
Cysteine and methionine metabolism	10 (12.7)	19 (22.1)
Glycine, serine and threonine metabolism	10 (12.7)	14 (16.3)
Histidine metabolism	4 (5.1)	4 (4.6)
Lysine biosynthesis	6 (7.6)	2 (2.3)
Lysine degradation	7 (8.9)	10 (11.6)
Phenylalanine metabolism	16 (20.2)	14 (116.3)
Phenylalanine, tyrosine and tryptophan biosynthesis	6 (7.6)	8 (9.3)
Tryptophan metabolism	8 (10.1)	12 (13.9)
Tyrosine metabolism	10 (12.7)	13 (15.1)
Valine, leucine and isoleucine biosynthesis	3 (3.8)	6 (7.0)
Valine, leucine and isoleucine degradation	18 (22.8)	15 (17.4)
Citrate cycle (TCA cycle)	19 (24.0)	29 (33.7)
Glycolysis/Gluconeogenesis	21 (26.6)	48 (55.8)
Pentose phosphate pathway	15 (19.0)	20 (23.2)
Pyruvate metabolism	20 (25.3)	21 (24.4)
Galactose metabolism	4 (5.1)	7 (8.1)
Fructose and mannose metabolism	12 (15.2)	18 (20.9)
Starch and sucrose metabolism	16 (20.2)	18 (20.9)
Sulfur metabolism	2 (2.5)	3 (3.5)

A list of some of the enzymes identified in several of these pathways is given in Table S3. Between parentheses are given the figures extrapolated to a common sample size of 10,000 cDNAs for both spruce and beech.

### Targeted annotation of the cDNAs

Our annotation effort concentrated on gene categories encoding proteins with biochemical activities directly linked to the breakdown of plant compounds and other complex nutrients (lignocellulose, chitin, proteins but also secondary metabolites) and polypeptides involved in the mobilisation of soil nutrients such as transmembrane transporters. This targeted annotation was essentially performed through sequence homology searches against specialized protein databases, which compile members of each of these different protein categories, or by using reference sequences when such specialized databases did not exist (in the case of phytases and dioxygenases). Results were further filtered manually and/or by using the MetaBioME database [24] to remove enzymes with no obvious links to the studied processes (such as for example intracellular proteases component of the proteasome).

This annotation strategy identified a large variety of enzymes involved in the above-mentioned processes including many different enzymes classes active on most plant cell wall polymers (cellulose, hemicellulose, pectin and lignin) but also on starch and chitin which, as a component of arthropods exoskeleton and fungal cell wall, represents an abundant nitrogen source in forest soils (Table 3). Altogether, Glycoside Hydrolases (GH CAZymes) represented between 0.5% (beech) and 0.8% (spruce) of all cDNAs. In addition we also identified a set of enzymes responsible for the breakdown of non-cell wall organic molecules such as different classes of proteases, a phytase, a cutinase and a putative carotenoid ester lipase (Table S4). These latter enzymes were all highly similar to known excreted fungal enzymes.

**Table 3:** An illustration of the diversity of potential plant cell wall and other polysaccharides (starch, chitin) degrading enzymes identified among the beech and spruce soil ESTs.

Category	Family	Potential enzyme activity (/substrate)^a^	beech	spruce
**CAZymes** **			**65** b **^,^** c **(82)**	**113** b **^,^** c **(131)**
	**CBM** ns	**Carbohydrate Binding Activity**	**3 (3.8)**	**10 (11.6)**
	**CE** ns	**Carbohydrate Esterases**	**3** c **(3.8)**	**9** c **(10.5)**
	CE1	xylan, pectin	1	1
	**GT** ns	**Glycosyltransferases**	**21 (26.5)**	**23 (26.7)**
	**PL** ns	**Polysaccharide Lyases**	**0 (0)**	**1 (1.2)**
	PL1	pectate lyase/pectin	0	1
	**GH** *	**Glycoside Hydrolases**	**38** c **(48.1)**	**70** c **(81.3)**
	GH5	Cellulase/cellulose	2	5
	GH6	endoglucanase/cellulose	1	0
	GH7	endoglucanase/cellulose	3	4
	GH9	endoglucanase/cellulose	1	0
	GH10	endo-1,4-β-xylanase/hemicellulose	0	1
	GH11	xylanase/hemicellulose	2	1
	GH12	endoglucanase/xyloglucan	0	1
	GH28	galacturonase/pectin	0	2
	GH13	α-amylase/starch	0	3
	GH15	glucoamylase/starch	1	1
	GH18	chitinase/chitin	3	6
	GH31	α-glucosidase/xyloglucan	0	1
	GH35	β-galactosidase/pectin	0	2
	GH44	endoglucanase/cellulose	0	1
	GH45	endoglucanase/cellulose	0	2
	GH51	α-L-arabinofuranosidase/pectin	0	1
	GH54	α-L-arabinofuranosidase/pectin	2	0
	GH61	Cu-metalloenzyme/cellulose	1	6
	GH76	α-1,6-mannanase/hemicellulose	0	2
	GH78	α-L-rhamnosidase/pectin	0	1
	GH93	exo-α-L-1,5-arabinanase/pectin	0	1
**FOLymes** *			**6 (7.6)**	**24 (27.9)**
	**LO** ns	**Lignin Oxidases/lignin**	**3 (3.8)**	**5 (5.8)**
	LO1	Laccase/Catechol oxydase	2	1
	LO2	peroxydase	1	1
	LO3	cellobiose dehydrogenase	0	1
	**LDA** **	**Lignin Degrading Auxiliary enzymes**	**3 (3.8)**	**19 (22)**
	LDA1	aryl-alcohol oxydase	0	3
	LDA3	glyoxal oxydase	0	7
	LDA5	galactose oxydase	0	1
	LDA6	glucose oxydase	0	1
	LDA7	benzoquinone reductase	3	5
	LDA8	alcohol oxydase	0	2

For the main categories, between parentheses are given the figures extrapolated to a common sample size of 10,000 cDNAs. Differences between Beech and Spruce were tested using Pearson Chi-square test;

**, *P*<0.01;

*, 0.01<*P*<0.05;

ns , not significant, *P*>0.05.

a according to data from the CAZyme database, [18], [29] and [67].

b Excluding CBMs which can be associated to other CAZYmes.

c These figures include all CAZYmes, CE or GH identified in the datasets; not just the families illustrated in this table.

Given that organic matter input in soil also results in the input of toxic secondary metabolites, we searched for enzymes classes potentially involved in their detoxification. While we only identified a single putative fungal dioxygenase (cathechol dioxygenase) among sequences from the spruce soil, we identified a total of 26 P450 monooxygenases belonging to 12 different families (as defined in the CYPED [25] database, Table S4). Among the CYPED homologous sequences, several did indeed contribute to the breakdown of toxic compounds such as benzoate or the flavonoid phytoalexin pisatin while others were part of biosynthetic pathways such as an O-methylsterigmatocystin oxidoreductase involved in aflatoxin production.

Besides their involvement in organic matter processing, most of these different enzymes are targets for the bioindustry. We therefore performed a global annotation of the environmental EST datasets with respect to their content in genes of biotechnological interest by blasting all sequences against the MetaBioME database which compiles sequences of “Commercially Useful Enzymes” (CUE). For both spruce and beech soils, more than 3% of the sequences returned a positive hit in this analysis and the two most represented CUE classes were the oxidoreductases and the hydrolases (Figure S3). About half of these sequences were from fungi (Figure S3).

Both spruce and beech soil metatranscriptomes included significant proportions (6.2 and 4.8% respectively; E-value threshold of 10^−6^) of putative membrane transporters as identified by blasting all cDNA sequences against the Transporter Classification Database (TCDB; Table 4) [26]. The most abundant category of fungal transporters involved in the assimilation of soil nutrients was the sugar porter one (T.C. 2.A.1.1) implicated in both hexoses (eg glucose, galactose, mannose) and pentoses (eg arabinose, xylose) assimilation, followed by the different families participating to amino acid and phosphate uptake. Concerning inorganic nutrients, besides phosphate transporters, we also identified ammonium transporters (T.C.1.A.11.) but neither nitrate (T.C.2.A.1.8) nor sulfate (T.C.2.A.53.1) ones.

**Table 4:** Diversity of fungal plasma membrane transporters, potentially involved in soil nutrient uptake, identified among the beech and spruce soil cDNAs.

TCDB^a^ family	description	beech	spruce
**Sugar transporters**			
2.A.1.1.-	Sugar porter family	1	31
**Amino-acid transporters**			
2.A.18.-	Amino acid/auxin permease (AAAP)	2	3
2.A.3.10.-	Amino Acid-Polyamine-Organocation (APC)	4	6(7)
**Peptide transporters**			
2.A.1.14.-	Anion:Cation Symporter (ACS)	1	0
2.A.67.-	Oligopeptide Transporter (OPT)	2	6
**Phosphate transporters**			
2.A.1.9.-	Phosphate: H^+^ Symporter (PHS)	2(3)	6
**Ammonium transporters**			
1.A.11.-	Ammonia Channel Transporter (Amt) Family	1	3
**Total transporters** **		**14**	**56**

Figures represent the No. of unique sequences after clustering; figures between brackets give the total No. of sequences before clustering. For the total number of transporters, differences between Beech and Spruce were tested using Pearson Chi-square test;

**, *P*<0.01.

a The Transporter Classification Database (http://www.tcdb.org/).

For most of the explored gene categories we consistently identified a higher number of homologous sequences in the spruce dataset compared to the beech one (Tables 3, S3 and 4). The most striking difference concerned the sugar porter family with 31 sequences in the spruce forest metatranscriptome (0.34% of the sequences) against only one in the beech forest data set (0.01%).

### Identification of full-length CAZymes

Twelve presumably full-length Carbohydrate Active Enzymes (CAZyme) encoding cDNAs, belonging to families CE1 (accession no. FR865947), GH5 (FR865941), GH7 (FR865943-4), GH11 (FR865942), GH45 (FR865945), GH61 (FR865936-40) and PL1 (FR865946), were identified and sequenced entirely. The longest cDNA clone was 1671 bp-long and encoded a putative GH7 cellobiohydrolase. Except for the CE1 family member, which is thought to be an intracellular enzyme with S-formyl glutathione hydrolase activity, all other 11 predicted protein sequences possessed a N-terminal signal peptide in agreement with the potential polysaccharide degrading activities of the corresponding enzymes.

The putative taxonomic/phylogenetic origin of these 12 cDNAs was assessed using Bayesian (MrBayes) and/or maximum likelihood (PhyML) phylogenetic analyses of the deduced amino acid sequences. Protein sequences used in the phylogenetic analyses included not only the CAZyme best BLASTX hits from GenBank nr database but also homologous sequences identified among GenBank non fungal ESTs in addition to the gene models predicted from 57 recently released fungal and non fungal (animals, choanoflagellate) genome sequences (Table S5).

Each of the 12 full-length environmental sequences were homologous to fungal sequences and to sequences from other taxonomic groups either closely related to the Fungi, such as the Metazoa (GH7 and GH45) or the choanoflagellates (CE1), or to more distantly related groups such as the Plantae (GH5, PL1) or the Bacteria (GH5, GH11 and PL1). With the exception of the Carbohydrate Esterase family 1 (CE1) genes, all other studied CAZyme genes were not present in all genomes analysed and, when present, frequently occurred as gene families from which between one and three members per species were used for the phylogenetic analyses.

**Figure 2:**
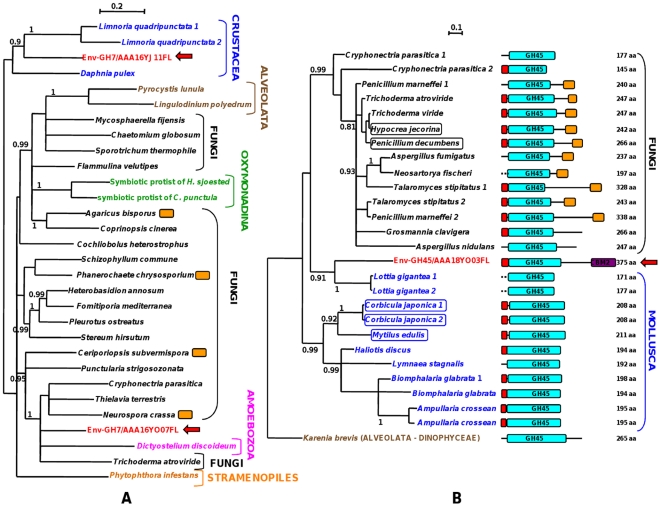
Putative phylogenetic origins of three environmental Glycoside Hydrolases belonging to families GH7 (A) and GH45 (B). Bayesian (MrBayes) phylogenetic trees include protein sequences from different taxonomic groups, each identified by a specific colour. Diagrams drawn to scale illustrate the modular structure of each of the different GH45 protein sequences. Red rectangles, potential signal peptides; blue rectangles, GH45 domains used for the phylogenetic analysis; orange rectangles, family one Carbohydrate Binding Modules (CBM1) characteristic of fungal GH45; purple rectangle, CBM2 module only found in the environmental sequence. Boxed species names indicate protein sequences for which an endoglucanase catalytic activity has been experimentally established [30]–[33]. Posterior branch probabilities above 0.8 are given; branches with less than 0.5 probability support were collapsed.

In many cases, sequences from one specific taxonomic group (eg Fungi or Bacteria) did not group together to form a single homogeneous and statistically well-supported clade (Figure 2, Figure S4). This could be due to an insufficient number of phylogenetically-informative characters in the sequences or to a complex evolutionary history of these gene families characterised by frequent gene duplications, gene losses and potential horizontal gene transfers between distantly related taxonomic groups [27]). *In fine*, only the phylogeny of the CE1 family fitted to the overall phylogeny of the Fungi [28] and the CE1 environmental sequence most likely originated from an Ascomycota Pezizomycotina fungal species (Figure S4D). Several other environmental sequences, including putative pectin lyase (PL1, Figure S4A), xylanase (GH11, Figure S4B), cellobiohydrolase (GH7, Figure 2B), endocellulase (GH45, Figure 2A) and members of the copper-dependent oxidase GH61 family [29] (Figure S4E), did not group with other referenced sequences to form statistically well supported phyla. This suggests that these sequences originated from fungal or non-fungal taxonomic groups distantly related to those from which homologous sequences have already been characterised. Percentages of identical amino acid positions between the environmental sequences and their closest relatives in the phylogenetic analyses (Figures 2 and S4) supported this hypothesis for several sequences. These percentages ranged from as low as 35–38% for two GH61 and the PL1 sequence to 67–70% for a GH7 and the CE1 sequences (Table S6).

Two of the environmental CAZymes potentially originated from the soil fauna. This was the case for one of the two analysed GH7 (putative cellobiohydrolase Env-GH7/16YJ11; Figure 2A) family members that grouped with the recently identified crustacean cellobiohydrolase polypeptides. This was also the case for the GH45 family member encoding a putative endocellulase (Figure 2B). This latter environmental sequence grouped with Molluscan GH45 sequences but differed from these sequences by the presence of a family two carbohydrate binding module (CBM2) at its C-terminal end (Figure 2B). Carbohydrate binding modules existed in several fungal GH45 proteins but they all belonged to the unrelated CBM1 family.

## Discussion

This report on the systematic sequencing of environmental eukaryotic cDNAs establishes the main features of this experimental approach. First, the specific analysis of polyadenylated mRNA allows access to the protein-coding gene pool of eukaryotes and efficiently counter-selects not only rRNA but also “non-eukaryotic” mRNA. This is in contrast to the studies published so far on the analysis of “global, essentially prokaryotic, metatranscriptomes” [34]–[38] which result in sequence datasets comprising as little as less than 1% [35]–[36] to often more than 50% of rRNA sequences despite the use of different protocols to eliminate these molecules [39], [40]. Furthermore, with less than 3% of cDNAs attributed to bacteria in both beech and spruce soil metatranscriptomes, polyadenylation of bacterial mRNA [41] does not seem to represent a challenge for the study of eukaryotic environmental mRNA. Moreover, the exact taxonomic origin of cDNAs attributed to bacteria should to be carefully evaluated as they may include genuine eukaryotic genes not yet identified in this taxonomic group such as bacterial genes recently acquired by eukaryotes by horizontal gene transfer; as for example recently suggested for nematodes carbohydrate active enzymes (CAZymes; [27]).

Secondly, among the cDNAs, between 52 and 60% have no homologs following BLASTX searches against the GenBank nr database. These figures, similar to the one reported for the Muskoxen rumen eukaryotic metatranscriptome [13], are higher than the percentages of orphan genes revealed in recently published genomic sequences from fungi and invertebrate phyla, that dominate the two studied soil eukaryotic communities. Figures vary widely from as low as roughly 5% for the entomopathogenic Ascomycota fungi *Metarhizum sp.* [42] to 20% for the symbiotic species *Tuber melanosporum* [43]. In the case of the insect *Pediculus humanus* [44] and the crustacean *Daphnia pulex* [45], two arthropods distantly related to other fully sequenced species from this phylum, the percentages of genes without homologs have been estimated to be of 10 and 36% respectively. Factors susceptible to accentuate the low percentage of cDNA taxonomic affiliation in eukaryotic metatranscriptomes include the presence of poorly studied protist groups in soils and also the large proportion of short sequences located at the 3′ end of the cDNAs which comprise untranslated 3′ UTRs. Future use of pyrosequencing or of other high-throughput sequencing technologies may increase the percentages of short reads, a factor that will be mitigated by a sharp increase in the number of sequences produced. All these figures must however be considered as transitory. Indeed, the current exponential increase in the number of available genome sequences in conjunction with a broader taxonomic sampling of the sequenced taxa, more representative of the soil biota, should have a direct and rapid positive impact on the taxonomic annotation of eukaryotic environmental sequences. More specifically it will allow us to carefully evaluate the contribution of protist taxa to the soil metatranscriptomes. Regarding functional annotation, better assignation will depend upon both (i) functional studies on individual unknown environmental sequences [46] and (ii) functional validation of unknown proteins from model organisms.

Despite the large proportion of housekeeping genes involved in basic cell maintenance mechanisms, both global (KEGG pathways) and targeted annotation identified a large variety of genes of interest in a context of soil ecosystem functioning and more specifically in the turnover of plant biomass and soil nutrient cycling and utilization. These nutrients encompass inorganic (ammonium, phosphates) and organic (aminoacids, peptides, phytate) forms of nitrogen and phosphorus, sulfates and simple sugars resulting essentially from either rhizodeposition or lignocellulose breakdown. We also noticed that genes representative of other key pathways such as the nitrate assimilation one (which comprises specific transporters, nitrate and nitrite reductases) were however missing in the datasets which could indicate that the corresponding nutrient represents a minor N source in the studied acidic forest soils.

Enzymes participating to plant cell wall deconstruction are of special interest not only from an ecological point of view to quantify and understand soil C turnover, but also because they are among the most widely used enzymes in the industry and are instrumental to the development of second generation biofuels. We identified among the beech and/or spruce soil cDNAs representatives of enzymes active on the main plant cell wall polymers: lignins, cellulose, hemicelluloses and pectins. Genes encoding carbohydrate active enzymes clearly outnumbered those encoding enzymes directly active on lignin although members of the three lignin oxidase families were identified in at least one of the two metatranscriptomes (Table 3). Glycoside Hydrolases (GH) encoding genes represented 0.46% (beech) and 0.78% (spruce) of the analyzed cDNAs. These figures are very similar to those reported for different “carbohydrate-adapted” metagenomes [18]. In a perspective of mining soil eukaryotic metatranscriptomes for such enzymes these values signify that the screening effort could be similar to the screening effort developed for metagenomes [14]. Additional sequencing effort as well as the study of replicate soil samples are nevertheless required before concluding on a potential effect of litter quality (beech *versus* spruce) on the relative abundance of transcripts related to plant cell wall degradation or nutrient assimilation (eg sugar transporters). It is indeed known that these two species differ with respect to the nature and proportions of polymers present in their cell walls [47], [48] and that spruce litter mineralizes more quickly compared to the beech one [49].

The use of Sanger sequencing of cloned cDNAs allowed us to easily identify several full-length genes among which several CAZyme ones. Phylogenetic analysis of the coded protein sequences suggests that many of them are distantly related to protein sequences present in databases. This further emphasizes the interest of eukaryotic metatranscriptomes as a source of novel enzymes with potentially interesting catalytic properties. This also indicates that organisms studied thus far at the molecular level in a context of plant cell wall deconstruction may not be representative of the organisms at work in the soil ecosystem. It is particularly interesting to consider that at least two out of the 12 full-length CAZymes (putative cellobiohydrolase and endocellulase) could originate not from fungi but from animals. A number of recent publications have indeed reported the existence of plant cell wall degrading enzyme genes in the genome of different groups of invertebrates which are represented among the soil fauna [27], [31], [33], [45], [50], [51]. This could indicate that in addition to their well recognized role in the fragmentation and mixing of plant litter, the soil fauna could also play a direct and potentially major role in the actual hydrolysis of plant carbohydrates in soils which adds up to hydrolysis by their gut microbiomes.

In conclusion, this first report of a eukaryote-specific analysis of soil metatranscriptomes highlights the potential of this experimental approach in two research fields. In the field of environmental biotechnology, eukaryotic metatranscriptomes represent diversified multigenome resources for many different gene categories used in the bioindustry. In this context, construction of environmental cDNA libraries whose potentially large inserts can be sequenced (this study) or directly expressed in heterologous hosts [20], [46], [52], [53] potentially represents the best experimental approach. In the field of environmental sciences, the study of eukaryotic metatranscriptomes will help us to reevaluate the contribution of the different soil eukaryotes to basic and essential soil processes such as organic matter degradation. It will also contribute to compare ecosystems which differ with respect to *i.e.* vegetation type, soil characteristics and climate and evaluate the relative contribution of these different variables on different soil processes. In this respect, high-throughput sequencing technologies should be implemented.

## Materials and Methods

### Study site and soil sampling

Soil samples were taken from an environmental research observatory forest site located in central France (Breuil-Chenue forest, 47°18′10″N, 4°4′44″E, 638 m above sea level). This site, initially covered by a mixed broadleaved tree forest (*Fagus sylvatica*, *Quercus sessiliflora*, *Betula verrucosa*, *Corylus avelana*) was clear-cut and replanted in 1976 by separate, single forest tree species stands, among which spruce (*Picea abies*) and beech (*Fagus sylvatica*). On July the 10^th^ 2007, 14 (spruce stand) and 16 (beech stand) soil samples (8 cm in diameter, 15 cm in depth) were collected along a systematic sampling grid. For each stand, the uppermost 3–7 cm thick organic matter-rich horizon of each core was sieved (2 mm mesh size) and mixed together (100 ml per core) to obtain a composite sample which was immediately frozen at −70 °C. Pedoclimatic parameters, soil characteristics and sampling strategy are detailed in Table S7.

### RNA extraction, cDNA libraries construction and sequencing

Total RNA (∼100 µg) was extracted from ∼90 g of each of the two composite forest soil samples as described in [20] and [54]. Polyadenylated mRNA were separated from non-polyadenylated RNAs (rRNAs, mitochondrial and prokaryotic mRNAs, tRNAs) by affinity capture on paramagnetic beads coated with poly-dT (Dynabeads Oligo (dT) kit, Dynal). Unbound non-polyadenylated RNAs were recovered by ethanol precipitation. Reverse transcription was carried out following the SMART cDNA Library Construction Kit instructions (Clontech), using between 90 (beech) and 210 ng (spruce) of purified mRNA. cDNA were further amplified by long distance PCR (LD-PCR) as described in the SMART cDNA Library Construction Kit, by using 20 and 24 PCR cycles for spruce and beech respectively. After size-fractionation to remove cDNA smaller than 400 pb (CHROMA SPIN-400 Columns, Clontech), the cDNAs were digested with *Sfi*1 and ligated into the *Sfi*1-digested pDNR-LIB plasmid vector (Clontech).

Each cDNA library was introduced in electro-competent *E. coli* cells (DH10B, Invitrogen) and 10,000 randomly selected cDNA clones were sequenced from their 5′ end using the M13-20 primer and Sanger chemistry (Genoscope sequencing centre, Ivry, France).

### 18S rDNA gene libraries construction and sequencing

350 (beech) and 730 (spruce) ng of soil non-polyadenylated RNA were reverse transcribed using 0.2 µg of random hexamers and 200 U of M-MuLV Reverse Transcriptase according to the manufacturer instructions (MBI Fermentas). A *ca* 560 bp-long fragment located at the 5′-end of the eukaryotic 18S rDNA gene was amplified by PCR using primers Euk1A (CTGGTTGATCCTGCCAG) and Euk516R (ACCAGACTTGCCCTCC) described by [55]. PCR mixtures (25 µl) contained 200 nM of each primer, 200 µM of each dNTP, 1 mM MgCl_2_, 0.25 mg.ml^−1^ of bovine serum albumin, 0.625 U of *Pfu* DNA polymerase, the appropriate buffer (Fermentas) and one tenth of the reverse-transcription mix. Amplification reaction started with an initial denaturation step of 3 min at 96°C, followed by 25 cycles comprising 45 sec at 96°C, 45 sec at 56°C and 2 min at 72°C, and finished with an elongation step of 5 min at 72°C. Amplification products of the expected size from 15 PCR tubes were pooled, isolated from an agarose gel (Nucleospin Extract kit, Macherey-Nagel) and ligated in the plasmid pCR-Blunt II-TOPO (Zero Blunt TOPO PCR Cloning kit, Invitrogen) that was used to transform electro-competent DH10B *E. coli* cells (Invitrogen).

For each rDNA library, the *ca* 560 bp rDNA inserts of 96 clones were entirely sequenced (Agowa Company, Berlin, Germany) using universal primer M13-20. Sequences were manually corrected and edited. BLASTn searches were performed against GenBank nr nucleotide database at NCBI (http://www.ncbi.nlm.nih.gov/) using default parameters except for word size which was set to 7. Sequences were analyzed with the rRNA Database Project CHECK_CHIMERA program (http://rdp8.cme.msu.edu/). Potential chimeras were further analysed by blasting separately the two dissimilar segments of the sequences against GenBank. Confirmed chimeras and artefacts were eliminated from the sequence datasets. All remaining sequences were submitted to EMBL and are available under accession numbers FN393180–FN393221 (beech) and FN393323–FN393380 (spruce).

### cDNA sequences cleaning and clustering

All cDNA sequences were trimmed using TIGR SEQCLEAN tool (http://www.tigr.org/tdb/tgi/software) with default parameters to eliminate poly-A sequences at the 3′end of cDNA, vector, adaptor and primer sequences and undetermined nucleotides. Sequences shorter than 100 nucleotides were eliminated. Contaminant ribosomal RNA sequences were identified by BLASTN analysis (cut-off threshold of E-value≤10^−10^, word-size = 11) of all sequences against LSUrdb and SSUrdb_SSURef100 libraries described in [38] (available at http://services.cbu.uib.no/supplementary/community-profiling/), as well as against NCBI-nr nucleotide database (looking for “ribosomal RNA” or “rRNA” in the title). The resulting “cleaned” sequence dataset was submitted to EMBL and is available under accession numbers FR706059–714330 (beech) and FR697056–706058 (spruce).

Cleaned sequences were clustered using Cd-hit (http://cd-hit.org, [56]) using a 90% identity threshold. The resulting clusters were used to perform a rarefaction analysis using S. Holland's Analytical Rarefaction version 1.3 software (http://www.uga.edu/strata/software/).

### Global cDNA sequences annotation

Cleaned cDNA sequences were queried (BLASTX) against NCBI nr protein database (http://www.ncbi.nlm.nih.gov/), using 2 different strategies, (i) Q-BLAST via BLAST2GO [57] with parameters, E-value≤10^−6^ and overlapping length >33% to the corresponding best hit and, (ii) NetBlast 2.2.22 (E-value≤10^−6^). As both analyses gave similar percentages of annotated cDNA sequences, we only considered the Q-BLAST results. In addition to raw BLASTX analyses, we also used BLAST2GO to perform Gene Ontology (GO, [58]), Enzyme code (E.C.) and InterPro (conserved patterns in sequences) annotations.

### Targeted annotation of cDNAs

Cleaned sequence datasets were searched for sequences similar to genes coding for organic matter degradation enzymes and for enzymes of biotechnological interest, using BLASTX searches (default parameters) against various specialized databases. Searches were made against Carbohydrate Active Enzymes (CAZy, http://www.cazy.org/; [59]); and Fungal Oxidative Lignin Enzymes (FOLy; http://foly.esil.univ-mrs.fr/; [60]) databases to find plant cell wall degradation enzymes. cDNA similar to cytochrome P450 potentially involved in detoxication of plant secondary metabolites were searched against CYPED database (downloaded on October 2009, http://www.cyped.uni-stuttgart.de/, [61]). Lipases/esterases and proteases were searched against the Lipases Engineering database (LED, downloaded on December 2009; http://www.led.uni-stuttgart.de/, [62]) and MEROPS (downloaded on January 2010, http://merops.sanger.ac.uk/index.shtml, [63]) respectively. Membrane transporters (for sugar, amino acids, oligopeptides and phosphate) were searched against the Transporter Classification Database (downloaded on April 2010, http://www.tcdb.org/, [26]). For other enzymes (phytases and dioxygenases), for which no specific databases exist, reference protein sequence were extracted from GenBank/EMBL/DDJB database and used as queries to perform a tBLASTn analysis (default parameters) against our cDNA libraries.

For all these specific analyses, the functional annotations obtained were further confirmed using a BLASTX analysis (default parameters) of the corresponding cDNAs against GenBank nr and/or Swissprot protein databases. We only retained cDNA sequences similar to the fungal targeted enzyme using a E-value threshold of 0.001. For cytochrome P450, lipases, proteases, phytases and dioxygenases, identified cDNAs were further filtered by BLASTX (default parameter, E-value≤10^−6^) against a curated database of commercially useful enzymes (MetaBioME, http://metasystems.riken.jp/metabiome/index.php, [24]) to retain only sequences similar to enzymes, which have known applications in industries. To identify full-length sequences, annotated cDNAs were translated and aligned with the closest protein sequences identified in Genbank/EMBL/DDJB to search for a putative start codon in the N-terminal region.

### Taxonomic annotation of rRNA and cDNA sequences

For PCR-amplified 18S ribosomal sequences, BLASTN searches (default parameters, except word size set at 7) were performed against the GenBank nr nucleotide database at NCBI (http://www.ncbi.nlm.nih.gov/). 18S sequences were then attributed to major eukaryotic phyla (listed in Figure 1 and Table S2) by performing phylogenetic analyses (BioNJ and PhyM) using for the sequence alignments the best Blast hits and a set of reference sequences for each of the different phyla. Analyses (sequence alignments and phylogenetic analyses) were performed using Seaview [64].

Using BLASTX output files, cDNAs were taxonomically assigned using MEGAN V 3.5 (Metagenome analyser, www-ab.informatik.uni-tuebingen.de/software/megan, [65]). This program uses the result of a BLAST comparison and assigns each read to a taxon at a specific taxonomic level. All parameters of MEGAN were kept at default values. For the cDNA taxonomic assignation, an additional analysis was performed by setting the “min support” option (the minimum number of sequence reads that must be assigned to a taxon) to one instead of 5 used as default parameter.

### Phylogenetic analyses

Protein sequences used for phylogenetic reconstructions were selected among BLAST results against different databases. Each of the environmental protein sequences were blasted against the GenBank/EMBL/DDJB nr protein database (BLASTX) and non human EST one (TBLASTN). Additional homology searches (BLASTX) were performed against the proteomes of fully sequenced organisms available through different websites (Table S5). Depending on the number and taxonomic diversity of the best BLAST hits, between one/two (individual genome sequences) and five (GenBank/EMBL/DDJB) protein sequences were selected for the sequence alignments.

Alignments were performed on the phylogeny.fr platform (www.phylogeny.fr; [66]) using MUSCLE (default parameters) followed by the selection of phylogenetically informative regions using GBlocks (low stringency parameters). The resulting cured alignment were used for phylogenetic analyses using MrBayes (less than 30 sequences) and/or PhyML. Parameters for Bayesian analyses were set as followed: number of substitution type, 6 (GTR); substitution model, WAG; rates variation across sites, invariable plus Gamma; analyses were run for 100,000 generations, sampling tree every 10 generations, burning of 100. PhyML was run on the Seaview platform [64] using default parameters except for the WAG substitution model.

## Supporting Information

Figure S1 **Clustering of the cDNA datasets.** (A) Rarefaction curves plotting the no. of cDNA sequences against the no. of clusters showing that most sequences are unique; (B) and (C), size distribution of the clusters showing that few of them contains more than 4 sequences.
(PDF)

Figure S2 **The five most represented Gene Ontology (GO) categories are the same for the spruce and beech datasets.**
(PDF)

Figure S3 **Global biotechnological potential of the cDNA datasets.** Distribution of cDNA sequences homologous to “Commercially Useful Enzymes” (CUEs) in the MetaBioME database according to enzyme activity (E.C. no.). Analysis was performed separately for all cDNAs and for those affiliated to the fungi (see Fig. 1).
(PDF)

Figure S4 **Putative phylogenetic origins of nine full-length environmental CAZymes.** Environmental sequences (in orange) belong to families PL1 (A), GH11 (B), GH5 (C), CE1 (D) and GH61 (E). Maximun likelihood (PhyML) phylogenetic trees include protein sequences from different taxonomic groups, each identified by a specific colour; red, Fungi Basidiomycota; pink, Fungi Ascomycota; orange, other Fungi; Black, Choanoflagellida; brown, Bacteria; green, Plantae. Correspondence between numbers and species names is given in Table S5. Black diamonds point to the sequences used for the calculation of the percentages of amino acid identity and similarity with environmental sequences (Table S6).
(PDF)

Table S1 **Characteristics of the PCR-amplified 18S rRNA sequence datasets.**
(PDF)

Table S2 **Taxonomic affiliation of the 18S rRNA and cDNA sequence datasets.**
(PDF)

Table S3 **An illustration of some of the key enzymes identified in major KEGG metabolic pathways relevant to either C, N or S metabolism.**
(PDF)

Table S4 **An illustration of the diversity of potential organic matter degrading enzymes, other than plant cell wall and polysaccharide active ones.**
(PDF)

Table S5 **Origin of the CAZyme sequences used in the phylogenetic analyses.**
(PDF)

Table S6 **Percentages of conserved, identical and similar, amino acid positions between the 12 full length environmental CAZyme proteins and one or two of their closest phylogenetically related neighbours.**
(PDF)

Table S7 **Stand and sampling characteristics.**
(PDF)
