# Supplementary figures and images for: Metatranscriptomics Reveals the Diversity of Genes Expressed by Eukaryotes in Forest Soils
Source: PLoS One. 2012 Jan 6;7(1):e28967. doi: 10.1371/journal.pone.0028967 (PMC3253082; doi:10.1371/journal.pone.0028967)

Figure S1

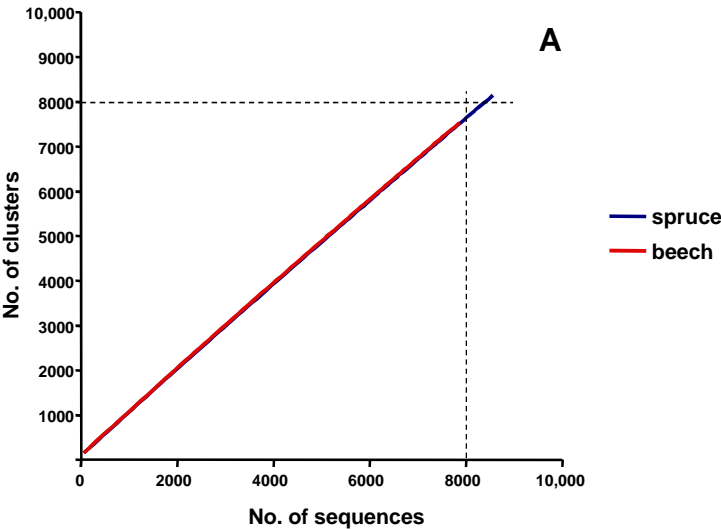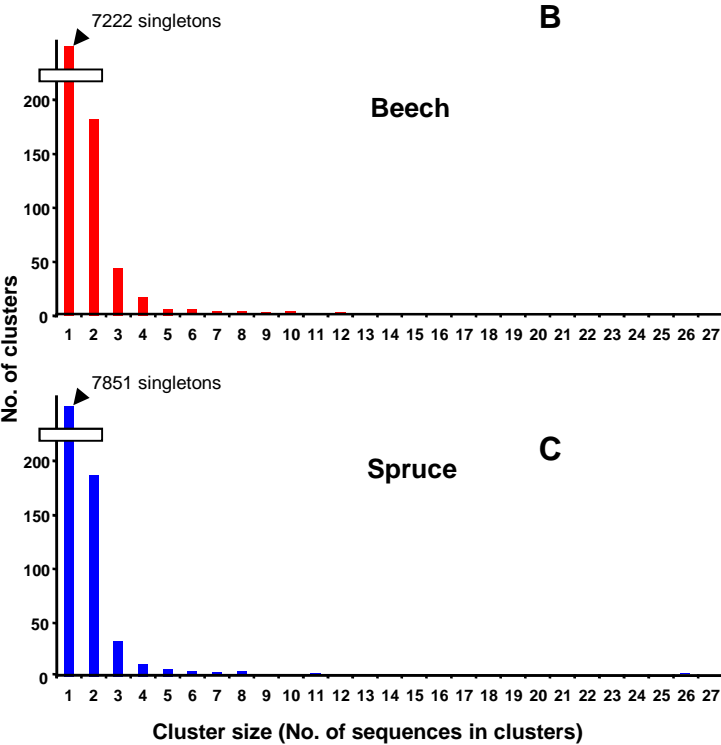

Supplement: Figure S1 — Clustering of the cDNA datasets. (A) Rarefaction curves plotting the no. of cDNA sequences against the no. of clusters showing that most sequences are unique; (B) and (C), size distribution of the clusters showing that few of them contains more than 4 sequences. (PDF) [file pone.0028967.s001.pdf]

**Figure S2**

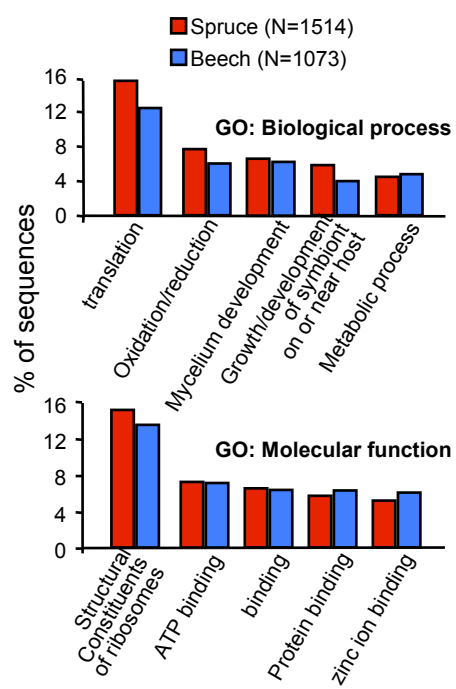

Supplement: Figure S2 — The five most represented Gene Ontology (GO) categories are the same for the spruce and beech datasets. (PDF) [file pone.0028967.s002.pdf]

Figure S3

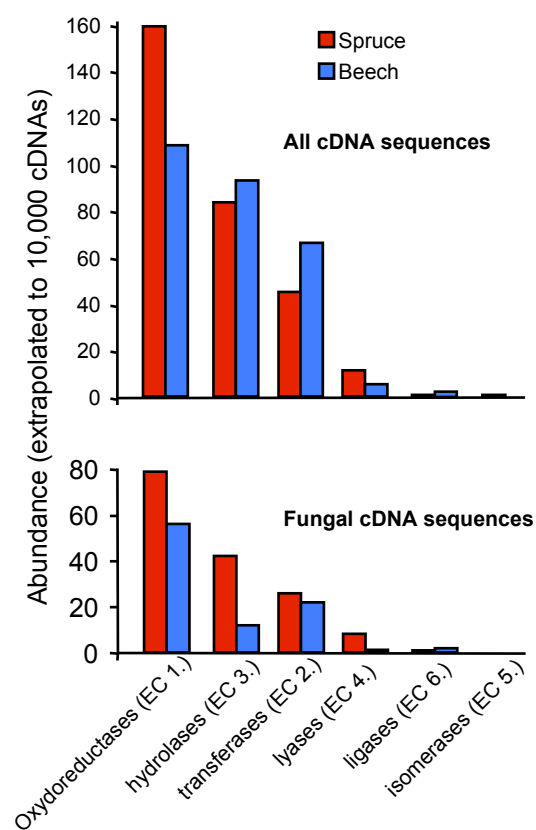

Supplement: Figure S3 — Global biotechnological potential of the cDNA datasets. Distribution of cDNA sequences homologous to “Commercially Useful Enzymes” (CUEs) in the MetaBioME database according to enzyme activity (E.C. no.). Analysis was performed separately for all cDNAs and for those affiliated to the fungi (see Fig. 1). (PDF) [file pone.0028967.s003.pdf]

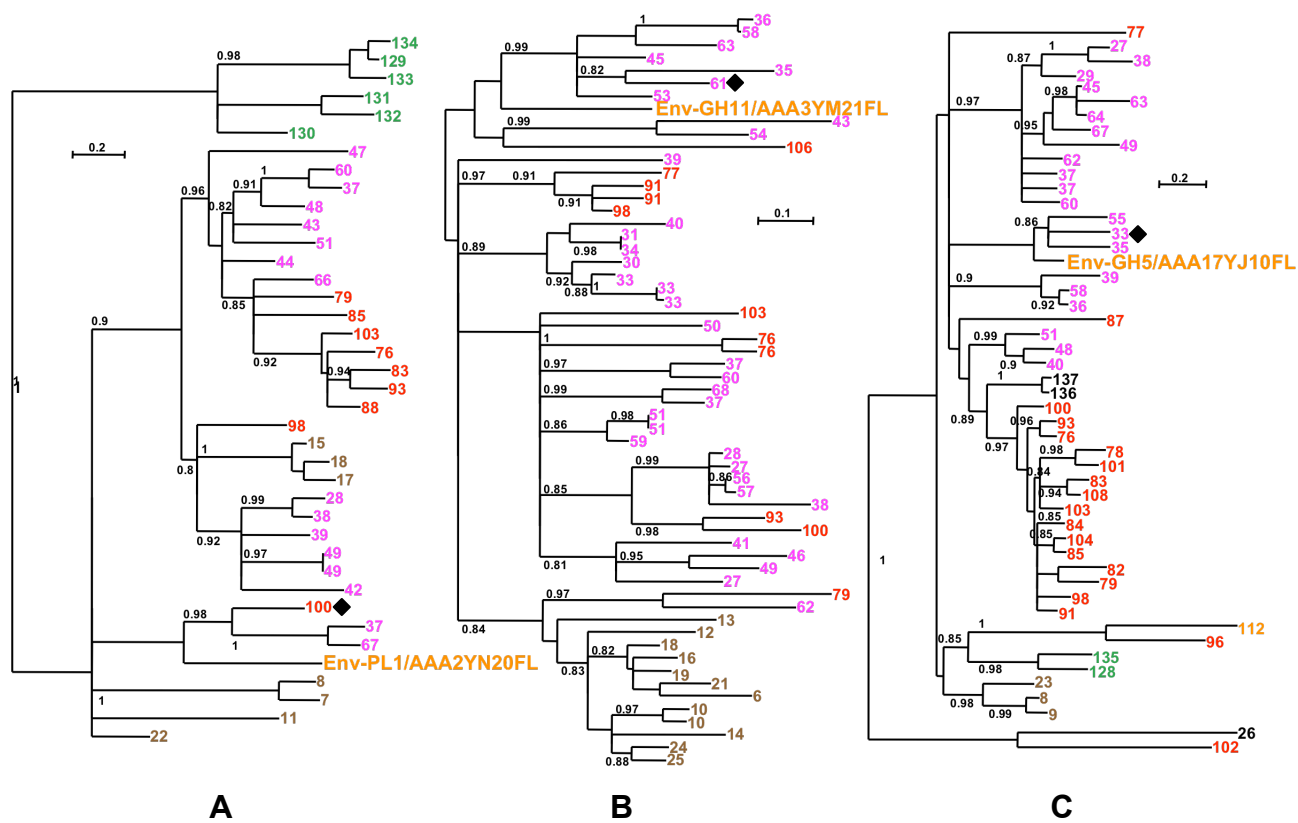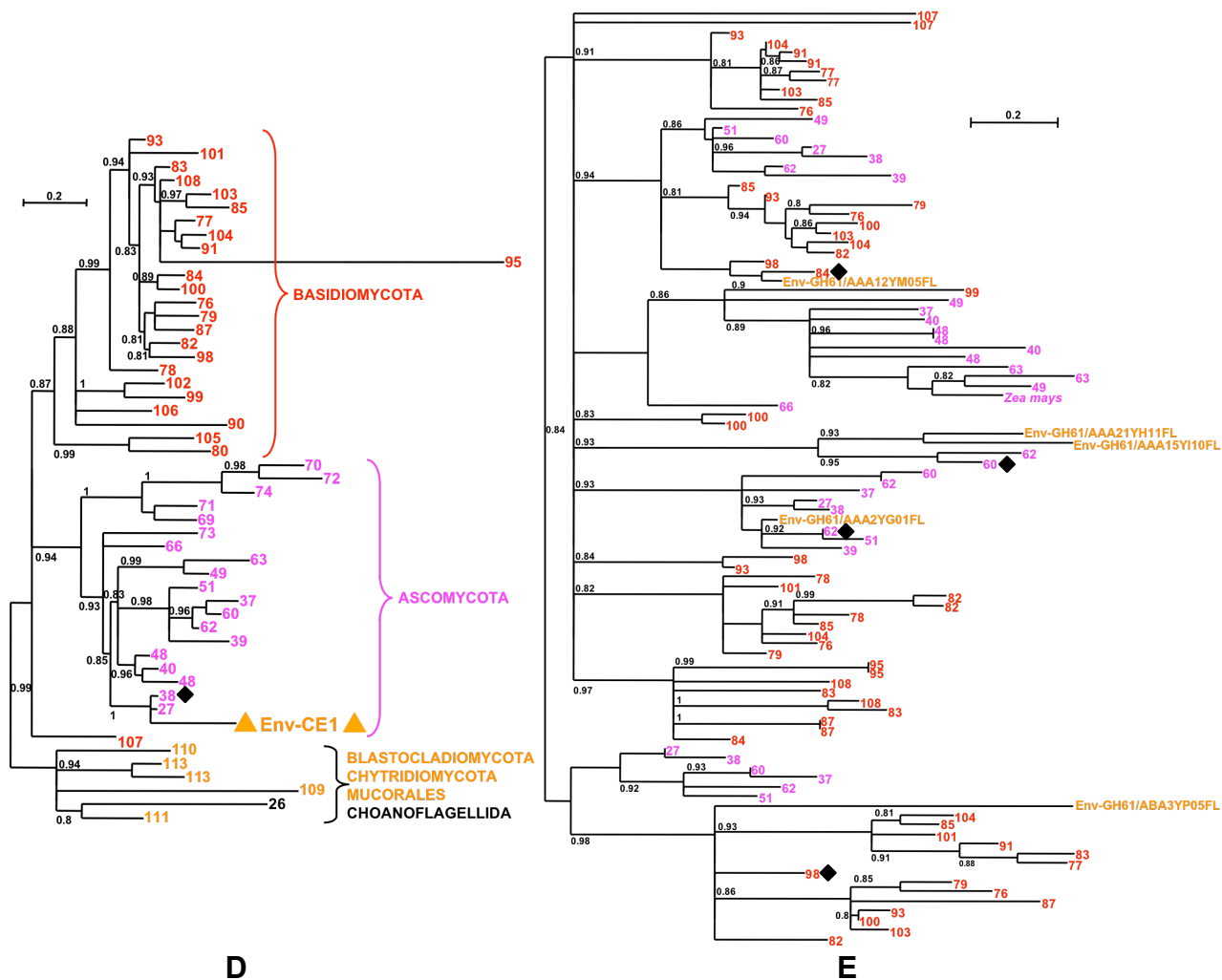

Supplement: Figure S4 — Putative phylogenetic origins of nine full-length environmental CAZymes. Environmental sequences (in orange) belong to families PL1 (A), GH11 (B), GH5 (C), CE1 (D) and GH61 (E). Maximun likelihood (PhyML) phylogenetic trees include protein sequences from different taxonomic groups, each identified by a specific colour; red, Fungi Basidiomycota; pink, Fungi Ascomycota; orange, other Fungi; Black, Choanoflagellida; brown, Bacteria; green, Plantae. Correspondence between numbers and species names is given in Table S5. Black diamonds point to the sequences used for the calculation of the percentages of amino acid identity and similarity with environmental sequences (Table S6). (PDF) [file pone.0028967.s004.pdf]
