# Supplementary material for: Metatranscriptomics Reveals the Diversity of Genes Expressed by Eukaryotes in Forest Soils
Source: PLoS One. 2012 Jan 6;7(1):e28967. doi: 10.1371/journal.pone.0028967 (PMC3253082; doi:10.1371/journal.pone.0028967)
Supplement: Table S1 — Characteristics of the PCR-amplified 18S rRNA sequence datasets. (PDF) [file pone.0028967.s005.pdf]

	spruce	beech
Total no. of 18S rRNA sequences	73	85
Putative chimeric sequences	4	23
“good quality” 18S rRNA sequences	60	42
No. of MOTUs (97 % nt identity threshold)	49	30

Table S1 Characteristics of the PCR-amplified 18S rRNA sequence datasets.

Putative chimeric sequences were identified using the CHECK_CHIMERA program
