# Supplementary material for: Metatranscriptomics Reveals the Diversity of Genes Expressed by Eukaryotes in Forest Soils
Source: PLoS One. 2012 Jan 6;7(1):e28967. doi: 10.1371/journal.pone.0028967 (PMC3253082; doi:10.1371/journal.pone.0028967)
Supplement: Table S2 — Taxonomic affiliation of the 18S rRNA and cDNA sequence datasets. (PDF) [file pone.0028967.s006.pdf]

	18S rRNA (PCR-amplified)	cDNA (MEGAN « default parameters »)	cDNA (MEGAN « stringent parameters »)
Fungi	24 / 17	2365 / 1410	2455 / 1490
Metazoa	21 / 13	369 / 384	458 / 502
Amoebozoa	2 / 2	64 / 56	70 / 74
Alveolata	2 / 2	19 / 5	32 / 20
Rhizaria	2 / 0	0 / 0	1 / 1
Heterokonta	0 / 1	8 / 5	12 / 11
Excavata	1 / 0	11 / 8	17 / 9
Plantae	6 / 7	169 / 84	195 / 114
Bacteria	0 / 0	71 / 114	164 / 212
Other Eukaryotes	0 / 0	0 / 10	13 / 18
Multiple affiliations	0 / 0	885 / 849	879 / 843

Table S2: Taxonomic affiliation of the 18S rRNA and cDNA sequence datasets from the spruce (first figures) and beech (second figures) forest soils. Putative protein coding cDNA sequences were annotated using two different settings of the MEGAN software. Using the "default parameters", the "Min. Support" value was set to five while it was set to one in the "stringent parameter" analysis.
