# Supplementary material for: Metatranscriptomics Reveals the Diversity of Genes Expressed by Eukaryotes in Forest Soils
Source: PLoS One. 2012 Jan 6;7(1):e28967. doi: 10.1371/journal.pone.0028967 (PMC3253082; doi:10.1371/journal.pone.0028967)
Supplement: Table S3 — An illustration of some of the key enzymes identified in major KEGG metabolic pathways relevant to either C, N or S metabolism. (PDF) [file pone.0028967.s007.pdf]

KEGG pathway	Enzyme	Beech	Spruce
**Nitrogen metabolism**		**13**	**17**
	glutamate synthase (NADPH)	0	1
	glutamate synthase (NADH)	0	1
	glutamate dehydrogenase	0	2
	glutamate dehydrogenase (NADP+)	6	2
	asparaginase	0	1
	glutamine synthase	4	6
	asparagine synthase (glutamine-hydrolysing)	0	1
**Alanine, aspartate and glutamate metabolism**		**19**	**26**
	glutamate dehydrogenase	6	2
	aspartate transaminase	2	3
	alanine transaminase	2	0
	glutamine synthase	4	6
	Asparaginase	0	1
**Arginine and proline metabolism**		**20**	**22**
	glutamate dehydrogenase	6	2
	aspartate transaminase	2	3
	glutamine synthase	4	6
	arginase	1	0
**beta-Alanine metabolism**		**6**	**13**
**Cysteine and methionine metabolism**		**10**	**19**
**Glycine, serine and threonine metabolism**		**10**	**14**
	threonine synthase	0	1
**Histidine metabolism**		**4**	**4**
**Lysine biosynthesis**		**6**	**2**
**Lysine degradation**		**7**	**10**
**Phenylalanine metabolism**		**16**	**14**
**Phenylalanine, tyrosine and tryptophan biosynthesis**		**6**	**8**
	aspartate transaminase	2	3
	anthranilate synthase	1	0
	tryptophan synthase	1	0
**Tryptophan metabolism**		**8**	**12**
**Tyrosine metabolism**		**10**	**13**
**Valine, leucine and isoleucine biosynthesis**		**3**	**6**
**Valine, leucine and isoleucine degradation**		**18**	**15**
**Citrate cycle (TCA cycle)**		**19**	**29**
	malate dehydrogenase	3	4
	isocitrate dehydrogenase (NAD+)	0	3
	isocitrate dehydrogenase (NADP+)	3	2
	pyruvate dehydrogenase (acetyl-transferring)	0	2
	succinate dehydrogenase	0	2
	citrate (Si)-synthase	2	2
	ATP citrate synthase	0	3
	phosphoenolpyruvate carboxykinase (ATP)	0	3

**Glycolysis / Gluconeogenesis**		**21**	**48**
	glyceraldehyde-3-phosphate dehydrogenase	1	10
	pyruvate dehydrogenase (acetyl-transferring)	0	2
	hexokinase	0	1
	fructose-bisphosphatase	0	2
	pyruvate decarboxylase	0	1
	phosphoenolpyruvate carboxykinase (ATP)	1	3
	triose-phosphate isomerase	1	5
	glucose-6-phosphate isomerase	0	2
	phosphoglucomutase	1	3
	acetate---CoA ligase	0	1
**Pentose phosphate pathway**		**15**	**20**
	glucose-6-phosphate dehydrogenase	1	1
	transketolase	3	3
	fructose-bisphosphatase	0	2
	fructose-bisphosphate aldolase	4	4
	phosphoglucomutase	1	3
**Pyruvate metabolism**		**20**	**21**
	malate dehydrogenase	3	4
	pyruvate dehydrogenase (acetyl-transferring)	0	2
	homocitrate synthase	1	1
	malate synthase	1	1
	phosphoenolpyruvate carboxykinase (ATP)	1	3
**Galactose metabolism**		**4**	**7**
	hexokinase	0	1
	beta-galactosidase	0	1
	phosphoglucomutase	1	3
	6-phosphofructokinase	1	0
**Fructose and mannose metabolism**		**12**	**18**
	hexokinase	0	1
	triose-phosphate isomerase	1	5
	aldehyde reductase	1	0
**Starch and sucrose metabolism**		**16**	**18**
	hexokinase	0	1
	alpha-amylase	0	1
	phosphoglucomutase	1	3
**Sulfur metabolism**		**2**	**3**
	adenylyl-sulfate kinase	1	0
	sulfate adenylyltransferase	1	1

Table S3: An illustration of some of the key enzymes identified in major KEGG metabolic pathways relevant to either C, N or S metabolism.
