# Supplementary material for: Metatranscriptomics Reveals the Diversity of Genes Expressed by Eukaryotes in Forest Soils
Source: PLoS One. 2012 Jan 6;7(1):e28967. doi: 10.1371/journal.pone.0028967 (PMC3253082; doi:10.1371/journal.pone.0028967)
Supplement: Table S4 — An illustration of the diversity of potential organic matter degrading enzymes, other than plant cell wall and polysaccharide active ones. (PDF) [file pone.0028967.s008.pdf]

Category	family	potential enzyme activity	beech	spruce
**Proteases^{ns} (MEROPS database)**			**11 (13.9)**	**13 (15.1)**
	A1	Aspartic endopeptidase	6	3
	G1	Acid protease	1	0
	M14	carboxypeptidase	0	1
	M24	Prolidase	0	1
	M28E	leucyl- aminopeptidase	1	0
	S8	subtilisin like serine protease	1	4
	S10	serine carboxypeptidase	0	1
	S53	tripeptidyl-peptidase	2	3
**Cytochrome P450 monooxygenases^{ns} (CYPED database)**			**9 (11.4)**	**17 (19.7)**
	102A	FAD-binding oxidoreductase	1	2
	116B	ferredoxin	1	0
	501A	lanosterol 14-alpha-demethylase	0	1
	505A	fatty acid hydroxylase	1	0
	512A	ent-kaurene oxidase	1	0
	52A	alkane hydroxylase	0	1
	55A	nitric oxide reductase	0	2
	57A	NA	0	2
	584G	alkane hydroxylase	3	2
	620H	O-methylsterigmatocystin oxidoreductase	1	2
	65AF	benzoate 4-monooxygenase	1	4
	704A	NA	0	1
**Lipases/esterases^{ns} (LED database)**			**0**	**2**
	abH03	Carotenoid ester lipase	0	1
	abH36	Cutinase	0	1
**Phytases^{ns}**	NA	phytase	**0**	**1**
**Dioxygenases^{ns}**	NA	Catechol 1,2-dioxygenase	**0**	**1**

Table S4: An illustration of the diversity of potential organic matter degrading enzymes, other than plant cell wall and polysaccharide active ones, identified among the beech and spruce soil ESTs. NA, not applicable.

For the main categories, between parentheses are given the figures extrapolated to a common sample size of 10,000 cDNAs. Differences between Beech and Spruce were tested using a Chi-square test; **, P<0.01; *, 0.01<P<0.05 ; ^{ns}, not significant, P>0.05.
