# Supplementary material for: Metatranscriptomics Reveals the Diversity of Genes Expressed by Eukaryotes in Forest Soils
Source: PLoS One. 2012 Jan 6;7(1):e28967. doi: 10.1371/journal.pone.0028967 (PMC3253082; doi:10.1371/journal.pone.0028967)
Supplement: Table S5 — Origin of the CAZyme sequences used in the phylogenetic analyses. (PDF) [file pone.0028967.s009.pdf]

No.	Taxonomy[a]	Species name	GH11	GH5	GH45	CE1	PL1	GH7	GH61	Source
**1**	Alveolata	*Karenia brevis*			+					GB
**2**	Alveolata	*Lingulodinium polyedrum*						+		GB
**3**	Alveolata	*Pyrocystis lunata*						+		GB
**4**	Amoebozoa	*Dictyostellium discoideum*						+		GB
**5**	Amoebozoa	*Polyspondylium pallidum*						+		GB
**6**	Bacteria	*Amycolatopsis mediterranei*	+							GB
**7**	Bacteria	*Bacillus clausii*					+			GB
**8**	Bacteria	*Bacillus licheniformis*		+			+			GB
**9**	Bacteria	*Bacillus pumilus*		+						GB
**10**	Bacteria	*Cellulomonas flavigena*	+							GB
**11**	Bacteria	*Dyctyoglomus thermophylum*					+			GB
**12**	Bacteria	*Kitasatospora setae*	+							GB
**13**	Bacteria	*Nesterenkonia xinjiangensis*	+							GB
**14**	Bacteria	*Paenibacillus curdlanolyticus*	+							GB
**15**	Bacteria	*Streptomyces bingchenggensis*					+			GB
**16**	Bacteria	*Streptomyces griseoflavus*	+							GB
**17**	Bacteria	*Streptomyces scabiei*					+			GB
**18**	Bacteria	*Streptomyces sp*					+			GB
**19**	Bacteria	*Streptomyces sp e14*	+							GB
**20**	Bacteria	*Streptomyces sp2*	+							GB
**21**	Bacteria	*Thermobispora bispora*	+							GB
**22**	Bacteria	*Thermonospora curvata*					+			GB
**23**	Bacteria	*Thermotoga maritima*		+						GB
**24**	Bacteria	*Xylanimicrobium pachnodae*	+							GB
**25**	Bacteria	*Xylanimonas cellulosilytica*	+							GB
**26**	Choanoflagellida	***Salpingoeca rosetta***	**0**	**+**	**0**	**+**	**0**	**0**	**0**	**BI**
**27**	Fungi/As/Pez	***Alternaria brassicicola***	**+**	**+**	**0**	**+**	**+**	**+**	**+**	**Jgi**
**28**	Fungi/As/Pez	*Alternaria sp.HB186*	+							GB
**29**	Fungi/As/Pez	*Aspergillus aculeatus*		+						GB
**30**	Fungi/As/Pez	*Aspergillus clavatus*	+							GB
**31**	Fungi/As/Pez	*Aspergillus flavus*	+							GB
**32**	Fungi/As/Pez	*Aspergillus fumigatus*			+					GB
**33**	Fungi/As/Pez	*Aspergillus nidulans*	+		+					GB
**34**	Fungi/As/Pez	*Aspergillus oryzae*	+							GB
**35**	Fungi/As/Pez	*Bispora sp*	+							GB
**36**	Fungi/As/Pez	*Botryotinia fuckeliana*	+							GB
**37**	Fungi/As/Pez	***Chaetomium globosum***	**+**	**+**	**0**	**+**	**+**	**+**	**+**	**Jgi**
**38**	Fungi/As/Pez	***Cochliobolus heterostrophus***	**+**	**+**	**0**	**+**	**+**	**+**	**+**	**Jgi**
**39**	Fungi/As/Pez	***Cryphonectria parasitica***	**+**	**+**	**+**	**+**	**+**	**+**	**+**	**Jgi**
**40**	Fungi/As/Pez	***Dothistroma septosporum***	**+**	**+**	**0**	**+**	**0**	**+**	**+**	**Jgi**
**41**	Fungi/As/Pez	*Fusarium oxysporum f sp. Lycopersici*	+							GB
**42**	Fungi/As/Pez	*Gibberella zeae*					+			GB
**43**	Fungi/As/Pez	*Glomerella graminicola*	+				+			GB

**44**	Fungi/As/Pez	*Grosmannia clavigera*			+					GB
**45**	Fungi/As/Pez	*Hypocrea jecorina*		+	+					GB
**46**	Fungi/As/Pez	*Magnaporthe oryzae*	+							GB
**47**	Fungi/As/Pez	*Marssonina brunnea*					+			GB
**48**	Fungi/As/Pez	***Mycosphaerella fijensis***	**?**	**+**	**0**	**+**	**+**	**+**	**+**	**Jgi**
**49**	Fungi/As/Pez	***Nectria haematococca***	**+**	**+**	**0**	**+**	**+**	**+**	**+**	**Jgi**
**50**	Fungi/As/Pez	*Neosartorya fischeri*	+							GB
**51**	Fungi/As/Pez	***Neurospora crassa***	**+**	**+**	**0**	**+**	**+**	**+**	**+**	**Jgi**
**52**	Fungi/As/Pez	*Penicillium decumbens*			+					GB
**53**	Fungi/As/Pez	*Penicillium marneffei*	+		+					GB
**54**	Fungi/As/Pez	*Phaeosphaeria nodorum*	+							GB
**55**	Fungi/As/Pez	*Phialophora sp*		+						GB
**56**	Fungi/As/Pez	*Pyrenophora teres*	+							GB
**57**	Fungi/As/Pez	*Pyrenophora tritici-repentis*	+							GB
**58**	Fungi/As/Pez	*Sclerotinia sclerotiorum*	+							GB
**59**	Fungi/As/Pez	*Sordaria macrospora*	+							GB
**60**	Fungi/As/Pez	***Sporotrichum termophile***	**+**	**+**	**0**	**+**	**+**	**+**	**+**	**Jgi**
**61**	Fungi/As/Pez	*Talaromyces stipitatus*	+		+					GB
**62**	Fungi/As/Pez	***Thielavia terretris***	**+**	**+**	**0**	**+**	**0**	**+**	**+**	**Jgi**
**63**	Fungi/As/Pez	***Trichoderma atroviride***	**+**	**+**	**+**	**+**	**0**	**+**	**+**	**Jgi**
**64**	Fungi/As/Pez	*Trichoderma longibraciatum*		+						GB
**65**	Fungi/As/Pez	*Trichoderma viride*			+					GB
**66**	Fungi/As/Pez	***Tuber melanosporum***	**0**	**0**	**0**	**+**	**+**	**0**	**+**	MW
**67**	Fungi/As/Pez	*Verticillium albo-atrum*		+			+			GB
**68**	Fungi/As/Pez	*Verticillium dahliae*	+							GB
**69**	Fungi/As/Sac	***Debaryomyces hanseni***	**0**	**0**	**0**	**+**	**0**	**0**	**0**	GL
**70**	Fungi/As/Sac	***Kluyveromyces lactis***	**0**	**0**	**0**	**+**	**0**	**0**	**0**	GL
**71**	Fungi/As/Sac	***Pichia stipitis***	**0**	**0**	**0**	**+**	**0**	**0**	**0**	**Jgi**
**72**	Fungi/As/Sac	***Saccharomyces cerevisiae***	**0**	**0**	**0**	**+**	**0**	**0**	**0**	GL
**73**	Fungi/As/Sac	***Yarrowia lipolytica***	**0**	**0**	**0**	**+**	**0**	**0**	**0**	GL
**74**	Fungi/As/Sac	***Zygosaccharomyces rouxii***	**0**	**0**	**0**	**+**	**0**	**0**	**0**	GL
**75**	Fungi/As/Tap	***Schizosaccharomyces pombe***	**0**	**0**	**0**	**0**	**0**	**0**	**0**	**BI**
**76**	Fungi/Ba	***Agaricus bisporus***	**+**	**+**	**0**	**+**	**+**	**+**	**+**	**Jgi**
**77**	Fungi/Ba	***Ceriporiopsis subvermispora***	**+**	**+**	**0**	**+**	**0**	**0**	**0**	**Jgi**
**78**	Fungi/Ba	***Coniophora puteana***	**0**	**+**	**0**	**+**	**0**	**+**	**+**	**Jgi**
**79**	Fungi/Ba	***Coprinopsis cinerea***	**+**	**+**	**0**	**+**	**+**	**+**	**+**	**Jgi**
**80**	Fungi/Ba	***Cryptococcus neoformans***	**0**	**0**	**0**	**+**	**0**	**0**	**0**	**Jgi**
**81**	Fungi/Ba	*Flammulina velutipes*						+		GB
**82**	Fungi/Ba	***Fomitiporia mediterranea***	**0**	**+**	**0**	**+**	**+**	**+**	**+**	**Jgi**
**83**	Fungi/Ba	***Fomitopsis pinicola***	**0**	**+**	**0**	**+**	**0**	**0**	**+**	**Jgi**
**84**	Fungi/Ba	***Gloeophyllum trabeum***	**0**	**+**	**0**	**+**	**0**	**0**	**+**	**Jgi**
**85**	Fungi/Ba	***Heterobasidion annosum***	**0**	**+**	**0**	**+**	**+**	**+**	**+**	**Jgi**
**86**	Fungi/Ba	*Irpex lacteus*						+		GB
**87**	Fungi/Ba	***Laccaria bicolor***	**0**	**+**	**0**	**+**	**0**	**0**	**0**	**Jgi**
**88**	Fungi/Ba	*Leucoagaricus gongylophorus*					+			GB
**89**	Fungi/Ba	*Melampsora larici populina*						+	0	**Jgi**

**90**	Fungi/Ba	***Melassezia globosa***	**0**	**0**	**0**	**+**	**0**	**0**	**0**	**Jgi**
**91**	Fungi/Ba	***Phanerochaete chrysosporium***	**?**	**+**	**0**	**+**	**0**	**+**	**+**	**Jgi**
**92**	Fungi/Ba	*Pleurotus florida*						+		GB
**93**	Fungi/Ba	***Pleurotus ostreatus***	**+**	**+**	**0**	**+**	**+**	**+**	**+**	**Jgi**
**94**	Fungi/Ba	*Polyporus arcularius*						+		GB
**95**	Fungi/Ba	***Postia placenta***	**0**	**+**	**0**	**+**	**0**	**0**	**+**	**Jgi**
**96**	Fungi/Ba	***Puccinia graminis***	**0**	**+**	**0**	**0**	**0**	**+**	**0**	**Jgi**
**97**	Fungi/Ba	*Puccinia graminis tritici*						+	0	**BI**
**98**	Fungi/Ba	***Punctularia strigosozonata***	**+**	**+**	**0**	**+**	**+**	**+**	**+**	**Jgi**
**99**	Fungi/Ba	***Rhodotorula graminis***	**0**	**0**	**0**	**+**	**+**	**0**	**+**	**Jgi**
**100**	Fungi/Ba	***Schizophyllum commune***	**+**	**+**	**0**	**+**	**+**	**+**	**+**	**Jgi**
**101**	Fungi/Ba	***Serpula lacrymans***	**0**	**+**	**0**	**+**	**0**	**0**	**+**	**Jgi**
**102**	Fungi/Ba	***Sporobolomyces roseus***	**0**	**+**	**0**	**+**	**0**	**0**	**0**	**Jgi**
**103**	Fungi/Ba	***Stereum hirsutum***	**+**	**+**	**0**	**+**	**+**	**+**	**+**	**Jgi**
**104**	Fungi/Ba	***Trametes versicolor***	**+**	**+**	**0**	**+**	**0**	**+**	**+**	**Jgi**
**105**	Fungi/Ba	***Tremella mesenterica***	**0**	**0**	**0**	**+**	**0**	**0**	**0**	**Jgi**
**106**	Fungi/Ba	***Ustilago maydis***	**+**	**0**	**0**	**+**	**0**	**0**	**0**	**Jgi**
**107**	Fungi/Ba	***Wallemia sebi***	**0**	**0**	**0**	**+**	**0**	**0**	**+**	**Jgi**
**108**	Fungi/Ba	***Wolfiporia cocos***	**0**	**+**	**0**	**+**	**0**	**0**	**+**	**Jgi**
**109**	Fungi/Blasto	***Allomyces macrogynus***	**+**	**0**	**0**	**+**	**+**	**0**	**0**	**BI**
**110**	Fungi/Chyt	***Batrachochytrium dendrobatidi***	**0**	**?**	**0**	**+**	**0**	**0**	**0**	**Jgi**
**111**	Fungi/Chyt	***Spizellomyces punctatus***	**0**	**+**	**0**	**+**	**0**	**0**	**0**	**BI**
**112**	Fungi/Mucorales	***Mucor circinelloides***	**0**	**+**	**0**	**+**	**0**	**0**	**0**	**Jgi**
**113**	Fungi/Mucorales	***Phycomyces blakesleanus***	**0**	**0**	**0**	**+**	**0**	**0**	**0**	**Jgi**
**114**	Fungi/Mucorales	***Rhizopus oryzae***	**0**	**+**	**0**	**0**	**0**	**0**	**0**	**BI**
**115**	Metazoa/Crustacea	*Daphnia pulex*						+		**Jgi**
**116**	Metazoa/Crustacea	*Limnoria quadripunctata*						+		GB
**117**	Metazoa/Mollusca	*Ampularia crossean*			+					GB
**118**	Metazoa/Mollusca	*Biomphalaria glabrata*			+					GB
**119**	Metazoa/Mollusca	*Corbicula japonica*			+					GB
**120**	Metazoa/Mollusca	*Haliotis discus*			+					GB
**121**	Metazoa/Mollusca	*Lottia gigantea*			+					**Jgi**
**122**	Metazoa/Mollusca	*Lymnaea stagnalis*			+					GB
**123**	Metazoa/Mollusca	*Mytilus edulis*			+					GB
**124**	Oxymonadina	*Pseudotrichonympha grassii*						+		GB
**125**	Oxymonadina	Symb protist of *Hodotermopsis sjoesedti*						+		GB
**126**	Oxymonadina	Symb protist of *Reticulotermes speratus*						+		GB
**127**	Oxymonadina	Symb protist of *Cryptocercus punctulatus*						+		GB
**128**	Plantae	*Arabidopsis lyrata*		+						GB
**129**	Plantae	*Carica papaya*					+			GB
**130**	Plantae	*Cupressus sempervirens*					+			GB
**131**	Plantae	*Lilium longiflorum*					+			GB
**132**	Plantae	*Oryza sativa japonica group*					+			GB
**133**	Plantae	*Picea sitchensis*					+			GB
**134**	Plantae	*Populus tremulaXtremuloides*					+			GB
**135**	Plantae	*Vitis vinifera*		+						GB

**136**	Stamenopiles/Oom	*Phytophthora infestans*	+		+	**Jgi**
**137**	Stamenopiles/Oom	*Phytophthora ramorum*	+			**Jgi**

Table S5: Origin of the CAZyme sequences used in the phylogenetic analyses (Fig. 2 and Fig. S4). Species No. are the No. used in the phylogenetic trees to identify the origin of the genes. Species in bold face characters are those for which the genomes were systematically searched for the presence of the different CAZyme categories. Sources of the data are as follow: GB, GenBank, (http://www.ncbi.nlm.nih.gov/genbank/); jgi, Joint Genome Institute (http://genome.jgi-psf.org/); BI, Broad Institute (http://www.broadinstitute.org/scientific-community/data?); Genolevure, GL (http://www.genolevures.org/); Mycorweb, MW (http://mycor.nancy.inra.fr/).

[a] As/Pez, Ascomycota/Pezizomycotina ; As/Sac, Ascomycota/Saccharomycotina ; As/Tap, Ascomycota/Taphrinomycotina; Ba, Basidiomycota; Chyt, Chytridiomycota; Oom, Oomycetes.
