# Supplementary material for: Metatranscriptomics Reveals the Diversity of Genes Expressed by Eukaryotes in Forest Soils
Source: PLoS One. 2012 Jan 6;7(1):e28967. doi: 10.1371/journal.pone.0028967 (PMC3253082; doi:10.1371/journal.pone.0028967)
Supplement: Table S6 — Percentages of conserved, identical and similar, amino acid positions between the 12 full length environmental CAZyme proteins and one or two of their closest phylogenetically related neighbours. (PDF) [file pone.0028967.s010.pdf]

CAZY family	Environmental sequence	Nearest neighbour[a]	Species No.[b]	% Identity / % Similarity
GH61	AAA2YG01FL	*Thielavia terrestris*	62	66 / 77
GH61	AAA12YM05FL	*Gloeophyllum trabeum*	84	59 / 71
GH61	AAA21YH11FL	AAA15YI10FL	NA	45 / 57
GH61	AAA21YH11FL	*Sporotrichum termophile*	60	38 / 53
GH61	AAA15YI10FL	*Sporotrichum termophile*	60	35 / 44
GH61	ABA3YP05FL	*Punctularia strigosozonata*	98	40 / 54
GH7	AAA16YO07FL	*Thielavia terrestris*	62	67 / 75
GH7	AAA16YO07FL	*Dictyostellium discoideum*	4	57 / 70
GH7	AAA16YJ11FL	*Daphnia pulex*	115	57 / 68
GH45	AAA18YO03FL	*Lottia gigantea*	121	42 [c] / 54 [c]
GH11	AAA3YM21FL	*Talaromyces stipitatus*	61	50 / 67
GH5	AAA17YJ10FL	*Aspergillus nidulans*	33	55 / 68
PL1	AAA2YN20FL	*Schizophyllum commune*	100	38 / 58
CE1	ABA10YA10FL	*Cochliobolus heterostrophus*	38	70 / 85

Table S6: Percentages of conserved, identical and similar, amino acid positions between the 12 full length environmental CAZyme proteins and one or two of their closest phylogenetically related neighbours as presented in the phylogenetic analyses (Figures 2 and S4). Percentages were deduced from global amino acid sequence alignments obtained using the Needleman-Wunsch algorithm as implemented at the ncbi web site (http://blast.ncbi.nlm.nih.gov/Blast.cgi?PAGE_TYPE=BlastSearch&PROG_DEF=blastn&BLAST_PROG_DEF=blastn&SHOW_DEFAULTS=on&BLAST_SPEC=GlobalAln&LINK_LOC=BlastHomeLink).

[a] Species with one of the “most related” protein sequence (Figures 2 and S4).

[b] As listed in Table S5

[c] alignment limited to 175 amino acid positions representing the conserved GH45 domain (see Figure 2).
