# Supplementary material for: Metatranscriptomics Reveals the Diversity of Genes Expressed by Eukaryotes in Forest Soils
Source: PLoS One. 2012 Jan 6;7(1):e28967. doi: 10.1371/journal.pone.0028967 (PMC3253082; doi:10.1371/journal.pone.0028967)
Supplement: Table S7 — Stand and sampling characteristics. (PDF) [file pone.0028967.s011.pdf]

Stand	beech	spruce
Sampling date	July 10 2007	July 10 2007
No. of soil samples	16	14
volume of samples (cm^3)	151-352	151-352
Bedrock	granite	granite
Mean annual temperature (℃)	6	6
Mean annual Rainfall (mm)	1400	1400
soil texture	sandy clayey	sandy clayey
soil water content (% at 15 cm)*	22.5	19.9
soil temperature (℃ at 15 cm)*	14.4	12.8
soil pH (water)	3.9	3.9
soil pH (KCl)	3.3	3.2
organic carbon (g/kg)	77.3	128
total nitrogen (g/kg)	4	6.4
C/N	19.2	20.2
% of organic matter	13.4	22.2
total calcium salt (g/kg)	<1	<1
phosphorus P_2O_5, Duchaufour method (g/kg)	0.151	0.173
phosphorus P_2O_5, Olsen method (g/kg)	0.02	0.032
total phosphorus P_2O_5 HF (g/kg)	1.22	1.35
cation exchange capacity cobaltihexamine (cmol+/kg)	8.61	9.53
proton exchange cobaltihexamine (cmol+/kg)	0.58	0.78
calcium Ca (cmol+/kg)	0.538	0.857
magnesium Mg (cmol+/kg)	0.236	0.444
sodium Na (cmol+/kg)	0.0374	0.113
potassium K (cmol+/kg)	0.315	0.439
iron Fe (cmol+/kg)	0.155	0.176
manganese Mn (cmol+/kg)	0.105	0.229

Table S7: Stand and sampling characteristics. Soil composition is given for the top organic horizons from which nucleic acids were extracted. * on the sampling day.
